# Prediction of Links and Weights in Networks by Reliable Routes

**DOI:** 10.1038/srep12261

**Published:** 2015-07-22

**Authors:** Jing Zhao, Lili Miao, Jian Yang, Haiyang Fang, Qian-Ming Zhang, Min Nie, Petter Holme, Tao Zhou

**Affiliations:** 1 Department of Mathematics, Logistical Engineering University, Chongqing, China; 2 CompleX Lab, Web Sciences Center, University of Electronic Science and Technology of China, Chengdu, China; 3 Department of Energy Science, Sungkyunkwan University, Suwon, Korea; 4 Big Data Research Center, University of Electronic Science and Technology of China, Chengdu, China

## Abstract

Link prediction aims to uncover missing links or predict the emergence of future relationships from the current network structure. Plenty of algorithms have been developed for link prediction in unweighted networks, but only a few have been extended to weighted networks. In this paper, we present what we call a “reliable-route method” to extend unweighted local similarity indices to weighted ones. Using these indices, we can predict both the existence of links and their weights. Experiments on various real-world networks suggest that our reliable-route weighted resource-allocation index performs noticeably better than others with respect to weight prediction. For existence prediction it is either the highest or very close to the highest. Further analysis shows a strong positive correlation between the clustering coefficient and prediction accuracy. Finally, we apply our method to the prediction of missing protein-protein interactions and their confidence scores from known PPI networks. Once again, our reliable-route method shows the highest accuracy.

Assume a link is missing from a network, link prediction aims to rank the best candidates of the vertex pairs for this missing link. Alternatively, assuming a network grows by links being added to it, link prediction can predict the next vertex pair to be connected by a link^1^. Thus, link prediction does not only help to find missing data in empirical networks, but also complements our understanding of the evolution processes of networks^2^,^3^,^4^. It has been an active subtopic of network science, in both more theoretical and applied directions. In general, topological features of the network and node attributes can be combined in the prediction algorithm. For example, in their work on predicting citations based on the citation networks, Popescul and Ungar considered not only topological characteristics of the network, but also the node attributes, such as authors, journal names and contents of the papers^5^. However, in many cases, it is difficult to get accurate information about the attributes of the nodes. For example, in online social networks, the information about users could either be inaccessible due to privacy policies or false. Therefore, many algorithms only use topological features. Some methods make use of functional outputs (of gene-regulatory networks)^6^ or address the slightly different problem of predicting link directions instead of link existence^7^.

There are two main classes of topology-based methods—similarity-based and likelihood-based methods^1^. A similarity-based algorithm assigns a similarity score for each pair of nodes and the unconnected node pair with higher score is supposed to have a higher probability of having a link. Authors have used local, global or quasi-local information to compute such similarity scores^8^,^9^,^10^,^11^,^12^. Likelihood-based algorithms presuppose some organizing principles of the network formation process and estimate the likelihood of any non-observed link under that assumption. Two popular algorithms of this type are the hierarchical structure model (HSM)^13^ and the stochastic block model (SBM)^14^,^15^,^16^,^17^.

Most previous studies in link prediction focus on unweighted networks. In recent years, a few works tried to extend the prediction algorithms from unweighted networks to weighted networks, typically by generalizing an unweighted similarity index to weighted networks^18^,^19^,^20^,^21^,^22^,^23^. Some strategies have been proposed to enhance the precision of weighted indices. For example, to emphasize the contributions of weak links^18^, to consider the authority of nodes^24^, or to integrate multiple indices^19^,^25^. However, few studies considered prediction of weights, which, in addition to the prediction of the existence of links, could be very valuable, perhaps especially so in biological networks. For example, protein-protein interactions (PPI) are identified from different types of experiments such as affinity chromatography^26^, co-immunoprecipitation^27^, GST pull-down^28^ and yeast two-hybrid^29^, with widely varying resolutions and accuracies. Data from such experiments varies much between different databases and authors. Some efforts have been made to integrate PPI data from different resources and then assign a confidence score to each pair of proteins^30^,^31^, resulting in a weighted PPI network. In predicting missing PPIs from such a network, predicting of confidence score of an interaction (i.e., the link weight) would be as important as predicting the existence of interaction. Lately, Aicher *et al.*^32^ proposed a likelihood-based algorithm which uses the weighted stochastic block model (WSBM) to predict the existence and weights of links in weighted networks.

In this study, we try to predict missing links and their weights using local similarity measures. Inspired by the solution of *the most reliable route problem* in communication networks^33^, we propose a method to generalize unweighted similarity indices. Assuming that the similarity index between two unconnected nodes reflects their interaction strength, and using the linear correlation between similarity scores and link weights in empirical networks, we set weights of missing links proportional to the similarity scores. We analyze our algorithm by measuring the accuracy of the weight prediction as the Pearson correlation coefficient and root mean-squared error (RMSE). We evaluate our method on seven empirical networks and identify the topological features that mostly affect the prediction accuracy. Finally, we apply the algorithm to predict protein-protein interactions in two human PPI networks and validate our output against another comprehensive PPI database.

## Materials and Methods

### Metrics

In this paper, we assume that weights are nonnegative, symmetric similarity weights measuring similarities or affinities between nodes. Often, larger similarity weights indicate closer relationships between nodes; hence such weights are positively correlated with the existence likelihoods of links. For example, weight of a collaboration network is the number of co-authored publications between two scientists, which is statistically correlated to the possibility that these two scientists will collaborate in the future^34^,^35^,^36^. In a protein-protein interaction network, weight is typically a confidence score of the interaction, representing the probability that the interaction occurs^37^. In a collaborative network of e-commerce users, weight characterizes the co-purchases between two users, which reflects the extent that the two users have similar shopping interests and thus may co-purchase more products in the future^38^. On the contrary, dissimilarity weights measure differences or distances between nodes. For instance, the weight of a road network can be Euclidean distance between neighbored intersections. For weight prediction on such networks our method would not perform well.

Given such a network *G*(*V, E, W*), where *V*, *E* and *W* are sets of nodes, links and weights, respectively, we want to find out its missing links (or links that may appear in the future) and predict their weights as well. To do this, for each pair of nodes without a link *x*, *y*∈*V*, we assign a similarity score *S*_*xy*_ to quantify the existence likelihood of the link (*x, y*). Then all unlinked pairs are ranked in the descending order of their scores, so that the links on the top can be considered as the ones with highest existence likelihoods.

To test the algorithm’s accuracy, we randomly divide the link set *E* into a training set *E*_*T*_ and a test set *E*_*V*_, such that E_*T*_∪E_*V *_=_* *_E and E_*T*_∩E_*V*_ = *ϕ*. We use two metrics, precision and AUC (the area under the receiver operating characteristic curve), for the accuracy measurement. Precision is the ratio of real missing links to predicted links. To be precise, if the top *L* links are considered predicted links while *L*_*r*_ of which appear in the test set, the precision is *L*_*r*_*/L*. AUC is a metric in the receiver operating characteristics (ROC) analysis^39^. Taking the top *L* links as predicted links, a ROC curve is obtained by plotting true positive rates (TPR) versus false positive rates (FPR) for varying *L* values. Good curves lie closer to the top left corner and the worst case is a diagonal line that represents a strategy of random guessing. Thus the total area under the ROC-curve (AUC) can measure the performance of the algorithm. Here we applied a simplified method to compute AUC value^1^. Specifically, at each time we randomly pick a missing link and a nonexistent link to compare their scores, if among *n* independent comparisons, there are *n’* times the missing link having a higher score and *n”* times they have the same score, the AUC value is


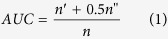


Different division of training and test set could result in different prediction accuracy. For the same network, the larger the training set, the smaller the test set. Usually, larger training set includes more information which makes the prediction easier. On the other hand, larger test set suggests higher background expectation of linkages. To make unbiased comparison between precisions under different sizes of training sets, we compute the odds ratio (OR) as follows^40^:


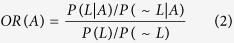


*OR*(*A*) represents the likelihood that a pair of nodes is linked given the result of a specific link prediction algorithm *A*. P(*L*|*A*) represents the probability of linkage between a pair of nodes conditioned on the result of algorithm *A*, i.e., the precision of algorithm *A*; and P(∼*L*|*A*) is the probability that a pair of nodes is not linked under the condition *A*. *P*(*L*) is the unconditional probability of linkage between a pair of nodes, which is the fraction of test set in the edges of the complement graph of the training set. According to Bayesian statistics, *OR*(*A*) is the likelihood of the linkage conditioned on the result of algorithm *A* and corrected for background expectations of linkages. Odds ratios greater than one indicate that algorithm *A* tends to link the node pairs, with higher values indicating more confident linkages.

According to our assumption, the score *S*_*xy*_ reflects the existence likelihood of a link between nodes *x* and *y*, while the weight *w*_*xy*_ measures pairwise similarity between *x* and *y*. Thus it is natural to assume that the similarity scores are proportional to the weights. To validate this hypothesis, we calculated the Pearson correlation coefficients between the vectors of similarity scores and actual link weights for the links in the test set and conducted statistical significance test. We obtained all the Pearson correlation coefficients larger than zero with all p-values less than 0.05, suggesting the linear correlation between similarity scores and link weights. Therefore, we can adjust the similarity scores to predict link weights. Specifically, denote the weighted adjacency matrix corresponding to *E*_*T*_ and *E*_*V*_ by *W*_*T*_ and *W*_*V*_, where *W*_*T*_ is known and *W*_*V*_ will be predicted. *S*_*V*_ are the similarity scores for links in *E*_*V*_. Next, we need to define a weight prediction function *F*(*W*_*T*_) so that the difference between *F*(*W*_*T*_) and *W*_*V*_ can be as small as possible.

Considering the above-mentioned linear correlation, we set *F* (*W*_*T*_) = *λ*·*S*_*V*_, where  *λ* is a scaling coefficient, which can be determined by solving the following optimization problem:


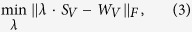


where ||.||F denotes the Frobenius norm, defined as the square root of the sum of the squares of the matrix’s elements^41^. We measure the accuracy of weight prediction by the Pearson correlation coefficient and the root mean-squared error (RMSE) between the vectors of predicted and known weights for links in *E_V_*.

### Similarity Indices

This study focuses on local similarity indices, which are designed based on the assumption that two nodes are more likely to have a link if they have many common neighbors. The assumption is supported by earlier empirical study on the evolvement of social networks^42^. Refs.^1^,^8^,^9^,^10^ systematically compare local similarity indices in unweighted networks and find that the so called Common Neighbors (CN), Adamic-Adar (AA) and Resource Allocation (RA) indices perform best. Thus we focus on these three measures whose definitions are as follows.

(i) CN index. The CN index simply counts the number of common neighbors between nodes *x* and *y* as:


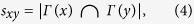


where Γ(*x*) is the set of neighbors of node *x* and 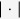 denotes cardinality of the set.

(ii) AA index^43^. This index depresses the contribution of the high-degree common neighbors by assigning larger weight to less-connected neighbors:


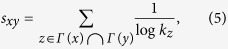


where *k*_*z*_ is the degree of node *z*.

(iii) RA index^9^,^44^. Similarly to AA index, RA index punishes the high-degree common neighbors, but to a higher extent, as


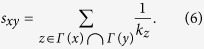


Previous studies extended similarity indices from unweighted networks to weighted networks by introducing the sum of weights of the two links (*z, x*) and (*z, y*), where *z* runs over all common neighbors of nodes *x* and *y*, as^18^,^22^:

(i) Weighted CN index (WCN):


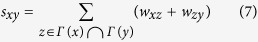


(ii) Weighted AA index (WAA):


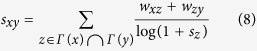


(iii) Weighted RA index (WRA):


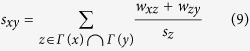


Here, *S*_*z*_ denotes the strength of node *z*, namely the sum of weights of its associated links, as


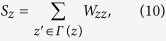


**Figure 1:**
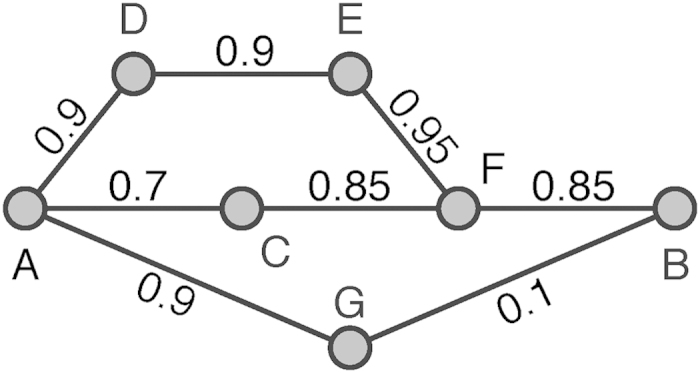
An illustration of a small communication network in which the weight of a link is its reliability. There are three routes from A to B in this simple communication network (A-D-E-F-B, A-C-F-B and A-G-B), in which the product of weights along links of the route A-D-E-F-B is the largest. Therefore, the most reliable route from A to B is A-D-E-F-B, whose reliability is about 0.58.

The most reliable route problem on a communication network asks for the most reliable route to transmit data packages from a source node to a destination node, which maximizes the probability that a package can reach the destination without being corrupted enroute. In this case, the communication network is represented as a weighted network, in which the weight of a link is the probability that this link is safe for data transmission. Usually, the reliability of each link is considered as independent. Thus the reliability of a route is the joint probability that all links along this route are intact, which is the product of the link weights^33^. Figure. 1 shows a simple example, where the route A-D-E-F-B is the most reliable route from A to B.

**Figure 2:**
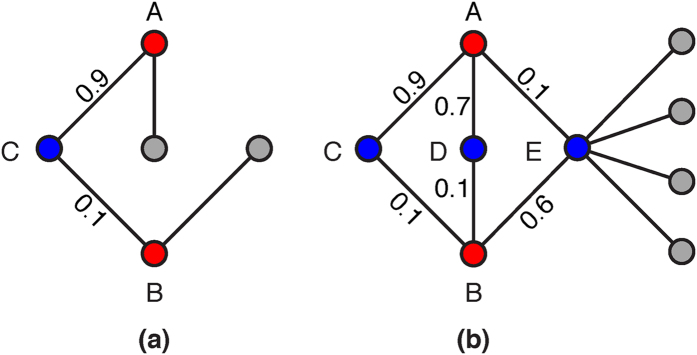
Visualized explanation of the reliable-route similarity scores of node pair (A,B) for two example networks (a) and (b). In network (a), under the condition that the existence likelihoods of links (A,C) and (C,B) are 0.9 and 0.1, respectively, the probability that A and B make a connection with the help of C (which may eventually result in a link between A and B) is the joint probability that (A,C) and (C,B) co-exist, i.e., 0.09. Although networks (a) and (b) have the same most reliable route A-C-B between A and B, network (b) has two other alternative routes A-D-B and A-E-B, constituting a parallel system. Thus the contributions of all independent route between A and B should be summed up to the similarity of (A,B). In addition, in network (b), connecting with many nodes other than A and B, node E’s contribution to the existence likelihood of link (A,B) should be smaller than that of nodes C and D. Contributions associated with such high-degree common neighbors are depressed in the rWAA and rWRA indices.

Assuming that weights of existing links are independent, it is reasonable to measure the similarity of a pair of unconnected nodes by the product of weights of local paths connecting them. Therefore, we define the so-called reliable-route weighted similarity indices as follows (Fig. 2 provides a straightforward explanation for this group of similarity indices):

(i) Reliable-route weighted CN index (rWCN):


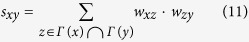


(ii) Reliable-route weighted AA index (rWAA):


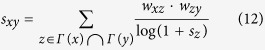


(iii) Reliable-route weighted RA index (rWRA):


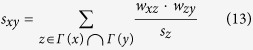


Since the weights in our work are analogous to link-existence probabilities, for networks whose weights do not lie in the range [0,1], before calculating reliable-route weighted similarity indices, we first normalize their weights by mapping to (0,1) through


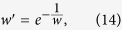


where *w* and *w’* denote the original and regulated weights, respectively. Since Eq. (14) is a one-to-one mapping, it is easy to extract the original weight *w* from the weight *w’*.

### Data Description

We use seven empirical weighted networks for this study, as follows.

- hsaPPI: a high-confidence protein-protein interaction network of human constructed from experimental biochemical co-fractionation data with overlap information derived from curated public databases and literature searches, in which the weight denotes the interaction confidence score^30^.
- Cel: the updated version of the neural network of *C. elegans*, in which nodes are neurons, edges are synaptic contacts between neurons, and the weight of a link represents the number of synapses between the corresponding neuron pair^45^.
- CGScience: the network of coauthorships between scientists publishing in computational geometry till February 2002, in which the link weight corresponds to the number of coauthored publications between two scientists. (See Pajek Datasets: http://vlado.fmf.uni-lj.si/pub/networks/data/collab/geom.htm.)
- Lesmis: the network of co-appearances of characters in Victor Hugo’s novel “Les Miserables”, in which the data on co-appearances were taken from^46^. Nodes represent characters and links connect any pair of characters that appear in the same chapter of the book. The link weights are the number of such co-appearances. (See Mark Newman’s network datasets: http://www-personal.umich.edu/~mejn/netdata/.)
- String: weighted human gene-association network constructed from the version 9.05 of the database STRING (Search Tool for the Retrieval of Interacting Genes/Proteins)^47^. STRING integrates both physical interactions and functional associations from numerous sources and associated each link with a probabilistic confidence score.

- Corum: a protein-protein interaction network of component proteins in human protein complexes collected by the database CORUM (Comprehensive Resource of Mammalian protein complexes)^48^. We downloaded the database CORUM in June of 2013, whose core data include 1343 complexes and 2314 component proteins. In this network, two proteins are linked if they appear in the same complex. The weights represent the number of shared complexes.

- String_Corum: a sub-network of String constructed by extracting the proteins in CORUM and their links from the network String.

See Table 1 for the basic topological measures of these networks. In Cel, CGScience, Lesmis and Corum, weights stand for the numbers of synapses, co-authors, co-appearances and shared complexes, respectively. As mentioned above, we will transform the weight *w* in these four networks to the range (0,1) by Eq. (14) before prediction.

## Results

### Accuracy of link and weight predictions

**Figure 3:**
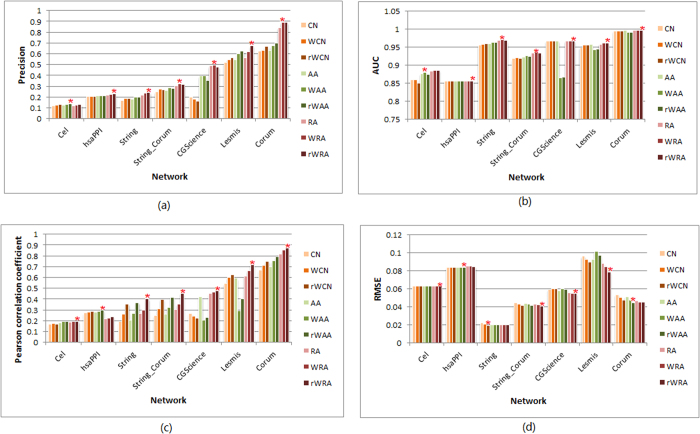
Accuracy of link and weight prediction for seven empirical weighted networks, where training and test set contain 90% and 10% of the original links, respectively. For each network, the star highlights the best prediction.

For each of the seven networks, we randomly split its links into a training and a test set, which contain 90% and 10% of the links, respectively. When calculating precision for link existence prediction, we set *L* equal to the size of the test set. Repeating this process 30 times, we obtained the average precision and AUC for link prediction as presented in Fig. 3a,b, respectively.

Figures 3a,b show that the best prediction results are achieved by weighted similarity indices, including WAA, rWAA, WRA and rWRA. This result suggests that for the class of weighted networks whose weights are defined by similarity between nodes, link weight is a very important indicator for measuring proximity between nodes. Thus, the accuracy of link predictions could be improved by taking weights of links into consideration. It can be seen that the weighted RA series perform best overall, which is consistent with the good performance of RA index in unweighted networks^1^. Especially, precisions and AUCs of rWRA method are either the highest or very close to the highest, showing the advantage of the reliable-route-based indices in link prediction.

We calculate Pearson correlation coefficients between the vectors of similarity scores and actual (normalized) weights for the links in the test set. As shown in Fig. 3(c), all the Pearson correlation coefficients are larger than zero (all the *p*-values are smaller than 0.05), suggesting the statistically significant positive linear correlation between similarity scores and link weights in all these cases. Since larger correlation coefficient indicates a more reliable dependence between weights and similarities and thus we can directly use Pearson correlation coefficients as the accuracy metric for weights. One can observe that all the highest accuracies in weight prediction are achieved by reliable-route weighted similarity indices, notably the rWRA index.

We also measure the accuracy of weight prediction by the root mean-squared error (RMSE) between the vectors of predicted and actual weights for links in the test set through solving the optimization problem defined in Eq. (3). The results are shown in Fig. 3(d). Similar to the Pearson correlation coefficients, the reliable-route weighted indices, especially the rWRA index, perform the best in weight prediction. Notably, both the metrics of Fig. 3c,d are in favor of rWRA.

### Robustness Analysis on the Size of Training Set

**Figure 4:**
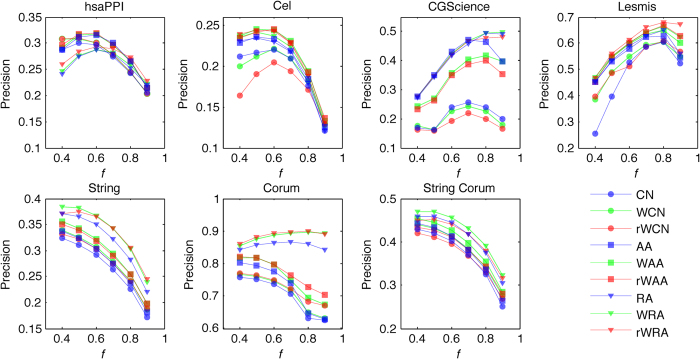
Precisions for link prediction under different approaches with different sizes of training sets (*f* symbolizes the fraction of links in training set).

**Figure 5:**
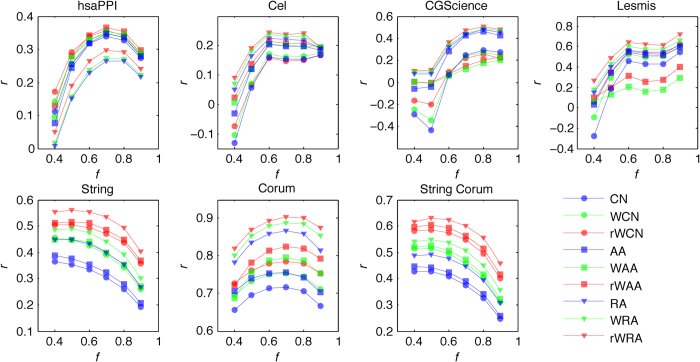
Pearson correlation coefficient (*r*) for weight prediction under different approaches with different sizes of training sets (*f* symbolizes the fraction of links in training set).

The accuracies for link and weight predictions for varying sizes of training sets (from 40% to 90%) are shown in Fig. 4 and 5, respectively. Each value of the accuracy is obtained by averaging over 30 implementations with independently random network divisions of the training set and test set. The number of predicted links, *L*, is always set as being equal to the size of the test set.

According to Fig. 4, with varying sizes of training set, prediction accuracies by reliable-route weighted indices (especially rWRA), are either the best or very close to the best. As for the accuracy of weight prediction, Fig. 5 shows that all the highest accuracies are achieved by reliable-route indices (especially rWRA) for different sizes of training set. These comparisons suggest the robust of our algorithms in both link and weight prediction.

**Figure 6:**
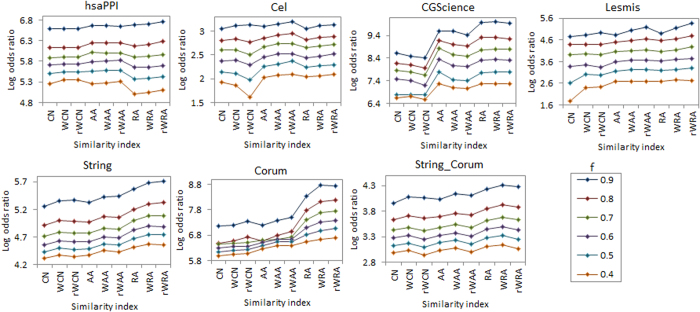
Log odds ratios for link prediction precisions under different approaches with different sizes of training sets (*f* symbolizes the fraction of links in training set).

Usually, larger training set contains more information which could make the prediction easier. However, Fig. 4 shows that the precisions do not always increase with the size of training set. This is caused by different prior linkage expectations of different sizes of test sets. Thus we correct the background expectation of linkages using the odds ratio defined in equation (2). As shown in Fig. 6, theconfidence of link prediction increases with the size of training set.

### Topological Analysis

In most cases, including weights can improve the prediction accuracy in our empirical data sets, however, networks CGScience and Lesmis are notable exceptions. For example, looking at Fig. 3(c) and Fig. 5, for the prediction accuracy of link weights on CGScience and Lesmis, WAA and rWAA are significantly lower than those of AA.

**Figure 7:**
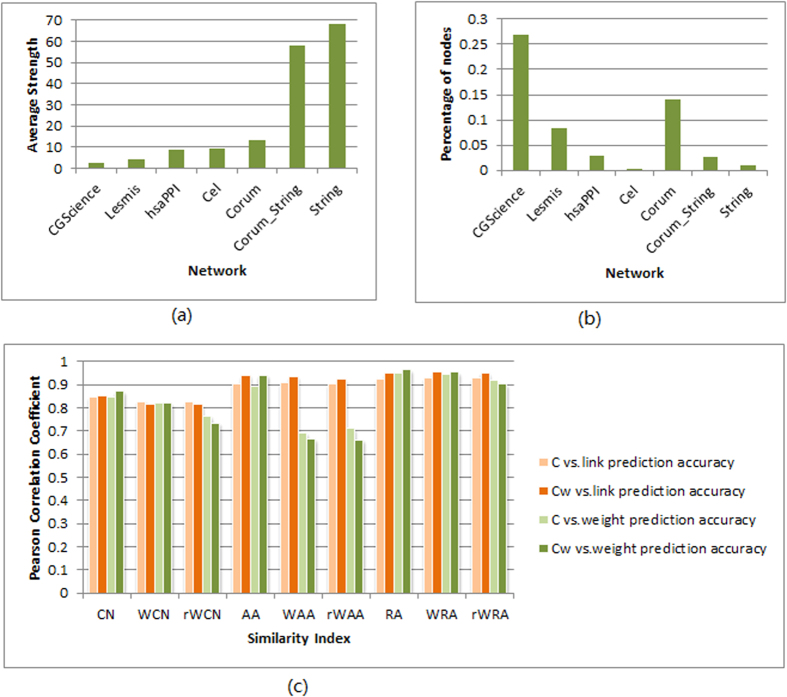
Network topologies features that may influence prediction accuracy. (**a**) Average node strength of networks; (**b**) Percentage of nodes punished by AA but rewarded by WAA and rWAA; (**c**) Correlation between accuracy of link or weight prediction and clustering coefficient (**C**) or weighted clustering coefficient (C_w_).

The AA index weighs nodes according to the logarithm of their degree (thus suppressing the role of hubs). When generalizing to weighted indices, instead of dividing log *k*_*z*_, such common neighbors are punished by dividing log(1 *+* *S*_*z*_). However, the average node strength values of networks CGScience and Lesmis are very small (see Table 1 and Fig. 7(a), implying that the contributions of high-degree common neighbors of these networks may not get enough inhibition in WAA and rWAA indices. In contrast to punishment to high-degree common neighbors, log *k*_*z*_ smaller than 1 could be considered as a reward in AA index because in this case node *z* only links to the node pairs under consideration. Similarly, in WAA and rWAA indices, log(1 *+* *S*_*z*_) smaller than 1 is a reward to the common neighbor *z*. When node strength is very small, the common neighbors punished in AA index are rewarded in WAA and rWAA indices, thus decreasing the prediction accuracy of WAA and rWAA methods. In Fig. 7(b) we show the percentages of such nodes in each network. It can be seen that this percentage is quite high in networks CGScience and Lesmis. Therefore, the low prediction accuracies in these two networks by WAA and rWAA might be caused by this aspect of node strength.

It can be seen that networks Corum and Lesmis get much higher prediction accuracies than other networks, which may be because of that these networks contains a plenty of cliques, as indicated by their large clustering coefficients for both unweighted and weighted versions (see Table 1), defined respectively as^49^,^50^:


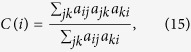


where *a*_*ij*_ equals to 1 when there is a link between node *i* and node *j*, else *a*_*ij*_ is zero. And


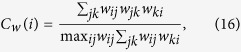


where *w*_*ij*_ represents the weight of link (*i,j*). The clustering coefficient of a network is the average clustering coefficient over all nodes. Since the current local indices only takes into account common neighborhoods of two nodes, it is straightforward to infer that the larger the clustering coefficient, the more accurate the prediction. From this respective, the poorer prediction performance on networks hsaPPI, String and Cel may largely own to their lower clustering. Indeed, as shown in Fig. 7(c), accuracies of link and weight predictions (*i.e.*, precisions in Fig. 3(a) and Pearson correlation coefficients in Fig. 3(c)) both exhibit a strongly positive correlation with the clustering coefficients. Especially, link prediction performance of AA and RA series shows a significantly higher dependence on clustering coefficient; meanwhile the weighted clustering coefficient gives a better characterization than the unweighted version. This indicates that depressing high-degree common neighbors could make triadic closure^49^ play more powerful role in link prediction task. As for weight prediction, the lower correlation extents of similarity scores by WAA, rWAA with clustering coefficients are caused by the special node strength feature of some networks, as we pointed out above.

However, weight prediction accuracy measured by RMSE does not show statistically significant dependence on clustering coefficient.

### Weight normalization functions

In our algorithm, we use a negative exponential function defined in Eq. (14) to normalize the weights of four networks whose weights are not in the area [0,1]. This is due to the feature of this function and the weights of four networks. With the growth of the positive independent variable *w*, the function in Eq. (14) grows quickly at first, then slows and finally levels off, approaching maximum upper limit 1. Similar to logistic function, this function can be applied to model saturation growth, such as biological population and product market growth. In the four networks, the link possibility exhibits a saturation growth with weights. For example, link weight of the CGScience network represents the number of co-authored publications between two scientists. In case two scientists co-authored enough papers, regardless the number is 50 or 100, the probability that they collaborate in the future is almost 1. Hence Eq. (14) could be fit for modeling linkage probability from weights of such networks.

**Figure 8:**
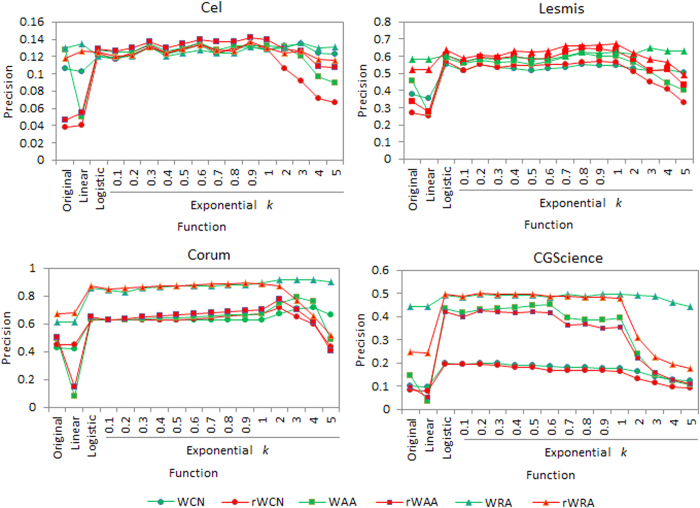
Precisions by weighted similarity indices for four networks under different weight normalizations, where the exponential function takes different parameter *k*, training and test set contain 90% and 10% of the original links, respectively.

**Figure 9:**
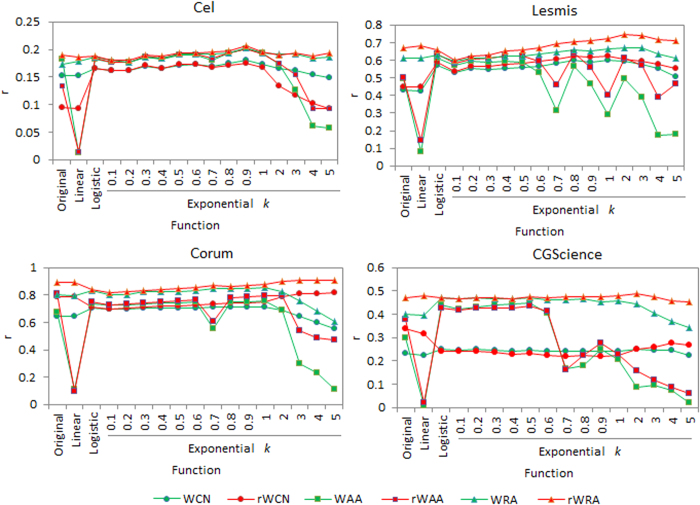
Pearson correlation coefficient (*r*) values by weighted similarity indices for four networks under different weight normalizations, where the exponential function takes different parameter *k*, training and test set contain 90% and 10% of the original links, respectively.

To verify the effectiveness of Eq. (14), we also normalize the weights by logistic function 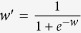, linear function 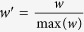 (max (*w*) is the maximum weight of the network), as well as negative exponential function 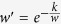 with different parameter *k*, respectively. Then for each of the four networks, we conducted link and weight prediction on the network with four types of weight, i.e., original weight, linearly normalized weight, logistic function normalized weight and exponential function normalized weight, respectively. Here we set the training set contain 90% links and *L* equal to the size of the test set. We repeat the computation for 30 times; average the prediction accuracies and show values of precision and Pearson correlation coefficient in Fig. 8 and 9. Figure. 8 shows that in most cases, weights normalized by logistic and exponential functions result in significantly higher precisions than the other two types of weights. We think this is because these two types of functions could model inherent linkage likelihood of node pairs from original weights of networks. In addition, the performance of exponential function with parameter *k* is robust when *k* varies between 0.1 and 1, further supporting the rationality and effective of our normalization method. Fig. 9 shows that for all the weight types, rWRA always performances best in weight prediction, confirming the robustness of this algorithm.

We also notice that for some weight types, such as weights normalized by linear function, exponential function with some specific parameters *k*, prediction accuracies by WAA and WAAr, especially weight prediction, are rather poor. We think the reason is the same as we discussed in the last section.

### Predicting protein-protein interactions

In cells, a protein usually collaborates with other proteins to carry out a particular cellular task. In other words, the other proteins that it interacts with often modulate its function and activity. Protein–protein interactions (PPIs) refer to such physical contacts between different proteins. Much of our knowledge of PPIs has been obtained by high-throughput experimental techniques such as affinity chromatography^26^ and yeast two hybrid^29^, as well as by manual curation of experiments on individual systems^51^. However, the currently known experimental results only reveal the tip of an iceberg of the actual existence of PPI links. For example, it is estimated that experimentally confirmed human protein-protein interactions account for only 0.3% of the actual existence^52^. Revealing the unknown part of these networks by experimental methods requires a lot of manpower, material and time. Therefore, it is highly desirable for developing computational methods for the prediction of largely unknown PPIs. A variety of computational approaches have been developed for the genome-wide inference of PPIs, which are based on similarity of protein biological attributes, such as sequence homology, gene co-expression, protein three-dimensional structural information, and phylogenetic profiles^53^,^54^. Here we investigate to what extent the topology-based link prediction methods could be applied in practice.

Of the empirical networks we study, hsaPPI and Corum are high-confidence protein-protein interaction networks of human beings, in which hsaPPI is constructed from the experimental biochemical co-fractionation data which overlap with information from curated public databases and literatures, while Corum represents experimentally derived co-complex memberships. The network String is constructed from the database STRING^47^, which is a comprehensive and reliable PPI database. Taking networks hsaPPI and Corum as input respectively, we predict the existence of protein-protein interactions and the confidence scores, and then use the network String to validate our predictions. That is, in this case, our training set includes total links in the input network, while the test set consists of overlapped links between String and the unconnected node pairs of the input network. When calculating precision for link existence prediction, we set *L* as 10% of the links in the input network.

**Figure 10:**
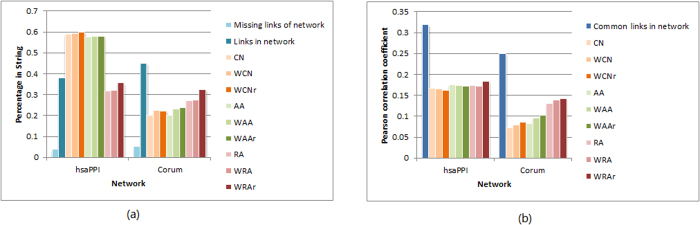
Validation of link and weight prediction for networks hsaPPI and Corum in the String network. (**a**) Percentages of top *L* predicted links in the String network, in comparison with those of actual links and unconnected node pairs in the input networks. (**b**) Pearson correlation coefficients between similarity scores and corresponding weights in the String network, in comparison with that between weights of common links in the String network and the input network.

Figure. 10(a) shows how many top *L* links predicted by the nine similarity indices that overlap with links in the String network. We compare the situations of actual links and unconnected node pairs of the input networks. First, note the small overlap of unlinked node pairs compared to the number of links in the input network, compared to these the overlap with String links is much (~8 times) larger. This is consistent with the high reliability of the STRING database. The top *L* predicted links have very large percentage of overlaps with String links, which is comparable with that of the actual links. Remarkably, the top *L* links predicted by methods of CN and AA series for network hsaPPI have even much higher percentage of overlap with String links than the actual links in the network. These results suggest high accuracy of these methods in practical prediction for PPIs. In addition, the highest precisions are achieved by rWCN and rWRA methods in network hsaPPI and Corum, respectively, confirming the good performance of reliable-route method.

Taking test set as that includes overlapped links between String and the unconnected node pairs of the input network, we then calculate Pearson correlation coefficients between the vectors of similarity scores and weights for the links in the test set. Statistical tests get all of the *p*-values smaller than 0.05, indicating statistically significant linear correlation between similarity scores of unconnected node pairs and weights of corresponding links in the String network. This suggests that the topological based methods are potentially applicable in the practical prediction of confidence scores for PPIs. Figure. 10(b) shows that the highest extents of positive correlation are achieved by rWRA method in both input networks, further confirming the good performance of reliable-route based method in weight prediction.

### Discussion and conclusion

This work aims to predict missing links and their weights using only local information, based on the assumption that two nodes are more likely to have a link if they have many common neighbors. We propose a “reliable-route method” to generalize local similarity indices from unweighted to weighted networks. Our experiments on real networks show that the resource allocation indices perform overall best both in link and weight prediction. In addition, we find that the accuracy of both link and weight prediction is positive correlated with the clustering coefficient, supporting the assumption underpinning the method of local similarity indices. Moreover, our results suggest that the WAA and rWAA indices may not suitable in networks with very small average node strength.

The prediction accuracy could be affected by the network background. For example, there are four networks whose nodes are human proteins. The network hsaPPI only includes high-confidence physical interactions between proteins. This is thus the sparsest network among the four. In comparison, the network Corum was constructed to represent theoretical links between component proteins of experimentally validated protein complexes, which represents a specific class of high-confidence protein-protein interactions, i.e., co-complex memberships. This network is a projection of a bipartite network between protein and complexes, and is therefore the most regularly organized and densely connected. The network String comprises functionally associated pairs, including physical interactions, co-expression, co-localization, forming complex, and participating same biological process. In fact, the database STRING is constructed by integrating data from different experiments, curated databases and literature mining. Therefore, String is the noisiest one and its links are built up by different organizing principles, which is usually not easy for link prediction algorithms. Due to the different backgrounds and corresponding structure features of these networks, when we use a part of link in the current network to predict the other part by repeatedly random sampling processes, prediction accuracies for hsaPPI and String are relatively poor while Corum is highest. However, as a practical application, when predicting missing PPIs in the current network, we use all links of hsaPPI and Corum as training set and String as test set. Due to its high extent of sparsity, hsaPPI exist much more missing links than Corum. This could be the reason that hsaPPI gets much higher prediction accuracy than Corum in this situation.

For most networks, there is significantly large improvement from the worst to the best accuracy for both link and weight prediction. However, networks Cel and hsaPPI are exceptions. The precisions of link prediction for these two networks are the lowest among all the networks, and there are very small increases from the worst to the best precisions. It would be interesting to investigate whether this is caused by an inherent feature of these networks, or the algorithm.

Although we discuss the weight of networks as if it is restricted to the probability of link existence, our method could be applied to a broader set of weighted networks where the weight represents some kind of “transitive” interaction strength. That is, if the indirect connection strength is strong (via paths of two or more links), then the direct connection strength (link weight) is probably also strong (which is the assumption behind some similarity measures^1^).

## Additional Information

**How to cite this article**: Zhao, J. *et al.* Prediction of Links and Weights in Networks by Reliable Routes. *Sci. Rep.* **5**, 12261; doi: 10.1038/srep12261 (2015).

## Figures and Tables

**Table 1:** Basic topological features of the networks we study.

	hsaPPI	Cel	CGScience	Lesmis	String	Corum	String Corum
|*V*|	2821	281	7343	77	16886	2314	2270
|*E*|	13880	2402	11898	254	1520927	34148	153788
<*k*>	9.84	17.1	5.29	6.59	180.14	29.51	135.49
*<S>*	8.469	9.57	2.55	3.94	68.43	13.22	58.15
*C*	0.169	0.346	0.486	0.573	0.301	0.747	0.371
*C*_*W*_	0.167	0.291	0.535	0.602	0.231	0.795	0.278
<*d*>	4.5	2.32	5.32	2.64	2.49	4.34	2.1
*r*	0.362	–0.136	0.243	–0.165	–0.0198	0.703	–0.0225
*H*	3.28	1.64	4.71	1.83	2.8	3.35	4.05

|*V*| and |*E*| are the number of nodes and links. 〈*k*〉 and 〈S〉 are the average degree and strength. *C* and *C*_*W*_ are the unweighted^49^ and weighted^50^ versions of clustering coefficient, respectively (see definitions in Eqs. (15) and (16)). 〈*d*〉 denotes the average distance, *r* indicates the assortativity coefficient [44], and *H* is the degree heterogeneity, defined as 〈*k*^2^〉/〈*k*^2^〉.
